# A rare presentation of primary leiomyosarcoma of ovary in a young woman

**DOI:** 10.3332/ecancer.2015.524

**Published:** 2015-04-21

**Authors:** J Vijaya Kumar, Anil Khurana, Paramjeet Kaur, Ashok K Chuahan, Sunita Singh

**Affiliations:** Pandit Bhagwat Dayal Sharma Post Graduate Institute of Medical Sciences, Rohtak, Haryana, India

**Keywords:** leiomyosarcoma, ovary, surgery, chemotherapy, radiation therapy, soft tissue sarcoma

## Abstract

Primary ovarian leiomyosarcoma is a very rare tumour which is most commonly seen in postmenopausal women. A case is described here involving a 30-year-old female who presented with pain in the abdomen. The patient underwent laparotomy with peritoneal lavage, bilateral salpingo-oophorectomy, and total abdominal hysterectomy. Microscopic and immunohistochemical findings established the diagnosis of primary ovarian leiomyosarcoma. Similar to sarcoma of other tissues, an adjuvant chemotherapy regimen consisting of vincristine, epirubicin, and cyclophosphamide was given for this present case. Six months after treatment completion, the patient is on regular follow-up and disease-free on clinical and radiological examination.

## Introduction

Sarcomas originating primarily in the ovary are rare and make up less than 2% of all ovarian malignancies [1]. Primary ovarian leiomyosarcomas are very rare tumours constituting less than 0.1% [2]. Because of their rarity, there are no large series of case reports in the literature and definitive treatment guidelines have not yet been developed.

## Case Report

**Figure 1a–b.**
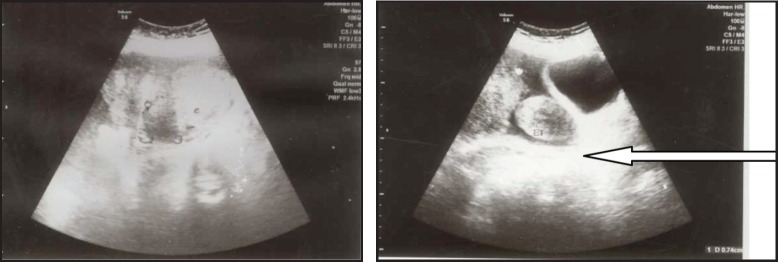
Ultrasonography of whole abdomen and pelvis showed a large mass occupying whole of pelvis just superior to fundus of uterus (not arising from the uterus).

**Figure 2.**
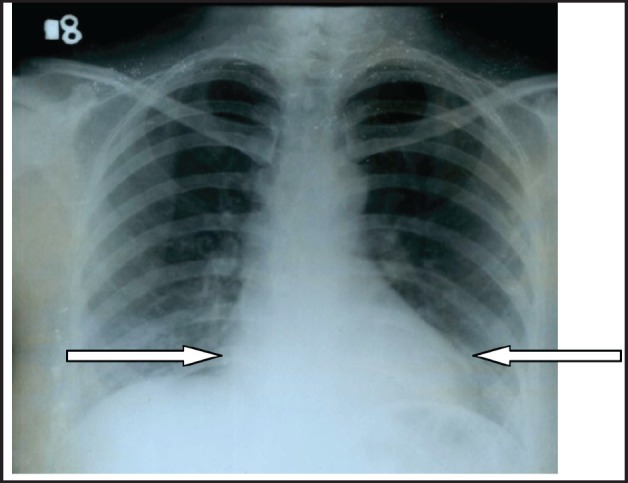
Chest x-ray PA digital view did not reveal any major abnormalities except for marginal increase of cardiac size in transverse diameter.

**Figure 3a–c.**
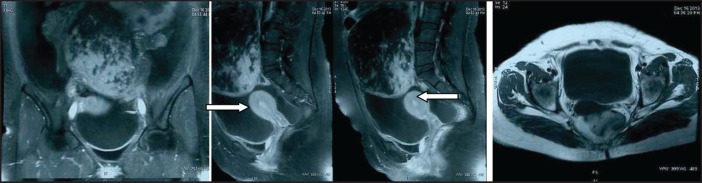
MRI of pelvis (coronal, sagittal, and transverse view) shows large oval well-defined mass lesion occupying whole of pelvis just superior to fundus of uterus (not arising from the uterus *indicated by arrow mark*).

**Figure 4a–b.**
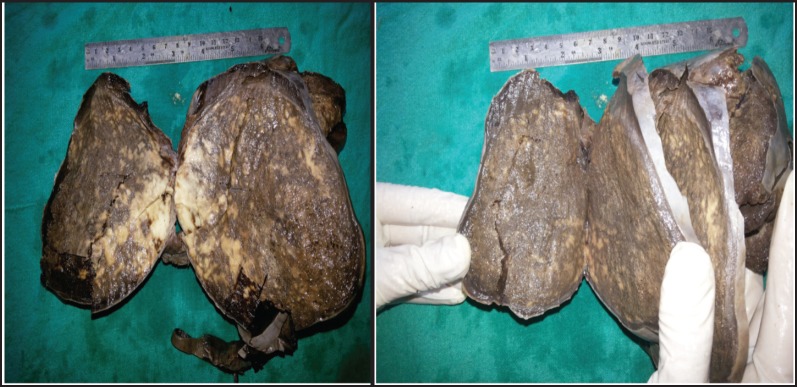
Cut section of right ovary specimen shows variegated appearance with focal areas of hemorrhage.

**Figure 5.**
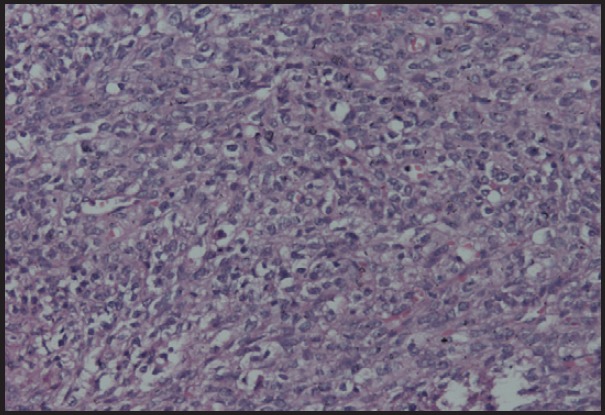
Photomicrograph showing oval to spindle cells with mild to moderate pleomorphism and high mitotic activity, H & E stain (200X).

**Figure 7a–c.**
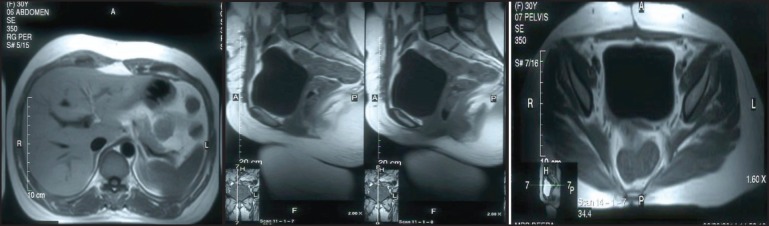
MRI of abdomen and pelvis (sagittal and transverse view) not showing any abnormalities after the completion of six cycles of adjuvant chemotherapy.

A 30-year-old lady presented with complaints of right lower abdominal pain since seven days which was insidious in onset and gradually progressive in nature. There was a history of difficulty in micturition and also a history of intermenstrual bleeding which were on and off for the past one year. There was no history of altered bowel habits. An abdominal examination did not reveal any abnormalities. On preoperative perivaginal examination when the cervix was pulled up, a mass was felt through the posterior fornix. Whereas on postoperative perivaginal examination, a small cystic mass was felt in the left fornix. Ultrasonography of the whole abdomen showed a large mass occupying the whole of the pelvis just superior to the fundus of the uterus arising from the right adnexa (not arising from the uterus) measuring 12.5 x 13.2 x 14.3 cc. There was also moderate free fluid seen in the peritoneal cavity with moving internal echoes (Figure 1a–b). The chest x-ray did not reveal any major abnormalities except for marginal increase of cardiac size in transverse diameter without any clinical cardiac abnormalities (Figure 2). The magnetic resonance imaging (MRI) of the pelvis showed a large oval, well-defined, altered signal intensity mass lesion measuring approximately 108 x 146 x 147 mm in size which occupied the lower abdomen and upper pelvis extending from upper L3 to S2 level superior to uterus. This lesion was displacing the adjacent bowel loops and was posteriorly abutting the iliac vessels and vertebrae. The lesion showed adequate heterogeneous enhancement more on the posterior and right lateral aspects along with enhancement of a tag-like structure and with mild heterogeneous enhancement in the centre. The left ovary was easily visible, measuring 19 x 15 mm, whereas the right ovary was small and ill-defined, measuring 10 x 16 mm. On the basis of these findings, a provisional diagnosis of leiomyoma, possibly pedunculated, was made (Figure 3a–c). However, in view of the ill-defined right ovary and moderate ascites further workup was suggested including ascitic fluid aspiration and cytology and CA-125 for the confirmation of the findings. The preoperative CA-125 level was 69.9 U/mL. The patient underwent laparotomy followed by right salpingo-oophorectomy and peritoneal lavage with perioperative findings revealing 15 cm x 15 cm ovarian mass with clotted blood (Figure 4a–b), areas of hemorrhage, and necrosis. The patient further underwent total abdominal hysterectomy with left salpingo-oopherectomy after the first course of adjuvant chemotherapy. Finally the patient was diagnosed as primary leiomyosarcoma of right ovary based on histopathological examination and immunohistochemistry (Figure 5). Immunohistochemistry of the tissue revealed positive for vimentin (Figure 6b), smooth muscle actin (Figure 6a), desmin, and it was negative for inhibin. Then the patient was planned for adjuvant chemotherapy with vincristine, epirubicin, and cyclophosphamide intravenously three weekly for six cycles. Six months after the treatment completion, the patient is on regular follow-up and is disease-free on clinical and radiological examination (Figure 7a–c).

## Discussion

Primary ovarian sarcomas are relatively uncommon representing less than 2% of all ovarian malignancies [1]. Primary leiomyosarcomas of the ovary are exceptionally rare comprising less than 1% of ovarian sarcomas [2]. Various other types of ovarian sarcomas include fibrosarcomas, endometrial stromal sarcomas, and rhabdomyosarcoma [3, 4]. Primary leiomyosarcoma of the ovary usually affects post-menopausal women with a few exceptions involving cases seen in younger women, as in the present case [1, 3].

Various pathogenesis related theories include malignant degeneration of smooth muscle tissue present in the ovary and malignant transformation of uterine leiomyoma to the ovary [4].

Most leiomyosarcomas present in advanced stages with vague symptoms of per vaginal bleeding, pelvic region pain, and altered bowel and bladder habits.

Grossly the tumour appears as solid masses with or without cystic degeneration which depends on the size of the tumour [5]. Microscopically tumour cells are uniform spindle-shaped cells which consists of abundant eosinophilic cytoplasm and elongated nuclei. These cells are arranged in a whorled pattern with areas of pleomorphism and necrosis [3].

Immunohistochemical markers used in the diagnosis of leiomyosarcoma of the ovary include desmin, vimentin, smooth muscle actin, S-100 [1, 6, 7]. It is also positive for oestrogen, progesterone receptors, p53, and bcl-2 in some cases [1, 7, 8].

Because of the rarity of the disease and late presentation, there is no established standard treatment. The main treatment modality is surgery which ranges from fertility sparing surgery to debulking surgery consisting of total abdominal hysterectomy, bilateral salpingooopherectomy, and excision of the tumour masses. Cytoreductive surgery can be performed during relapses and prognosis is good if the total tumour surgical resection is possible [1, 6, 9, 7, 10, 8, 11].

Even though chemotherapy and radiation therapy have been used in the adjuvant setting they do not provide any additional benefits [12, 13, 7]. The disease is associated with a poor prognosis [10].

## Conclusion

Leiomyosarcoma of the ovary is a rare malignancy. Because of the rarity of the disease and late presentation, there is no established standard treatment. The main treatment modality is surgery. Even though chemotherapy and radiation therapy have been used in the adjuvant setting often, there is no strong evidence of any additional benefit.

## Figures and Tables

**Figure 6a.**
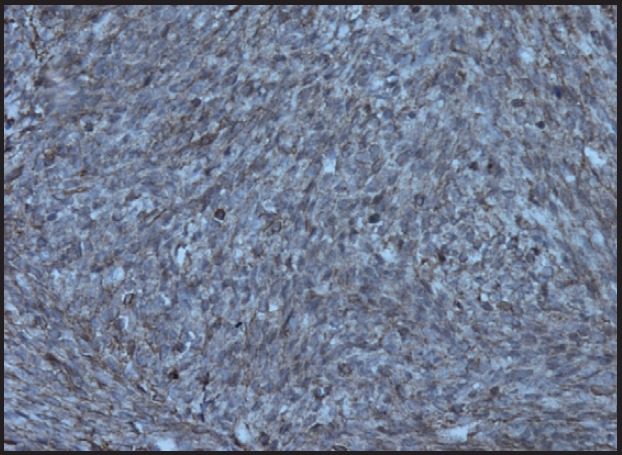
Photomicrograph showing tumour cells showing SMA positivity (200X).

**Figure 6b.**
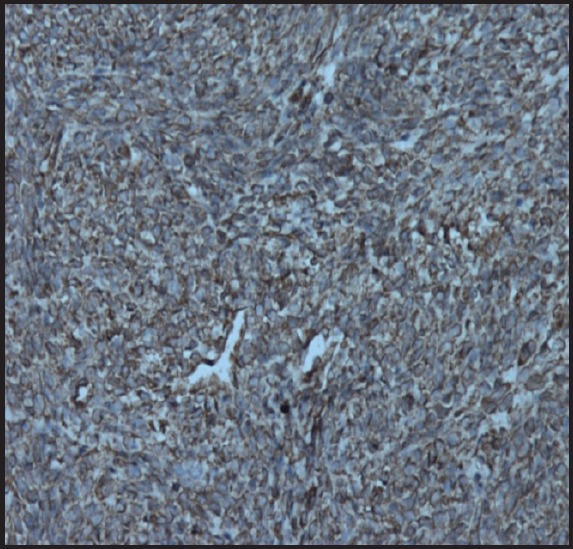
Photomicrograph showing tumour cells showing vimentin positivity (20X).
